# Unmasking of SGLT2 inhibitor associated ketoacidosis after initiation of mechanical ventilation with consecutive cardiopulmonary resuscitation and extracorporeal life support: A case report

**DOI:** 10.1097/EA9.0000000000000097

**Published:** 2026-02-20

**Authors:** Johannes Nienhaus, René M’Pembele, Detlef Kindgen-Milles, Hannan Dalyanoglu, Sebastian Roth

**Affiliations:** From the Department of Anesthesiology, University Hospital Duesseldorf, Germany (JN, RMP, DK-M, SR), the Department of Cardiac Surgery, University Hospital Duesseldorf, Germany (HD)

## Abstract

Sodium-glucose cotransporter 2 inhibitors (SGLT2i) are widely used for the treatment of type 2 diabetes, heart failure and chronic kidney disease due to their proven cardiovascular and renal benefits. However, they carry the risk of SGLT2i associated ketoacidosis, a rare but potentially life-threatening complication that can present without marked hyperglycaemia, often leading to delayed recognition. Although guidelines currently recommend discontinuing SGLT2i perioperatively and during acute illness, recent retrospective studies have questioned this approach, suggesting that perioperative continuation may not increase the incidence of SGLT2i associated ketoacidosis and could improve outcomes. While this trend is acknowledged, the present case highlights the dangers of continuing SGLT2i therapy in the intensive care setting with a special focus on the initiation of mechanical ventilation and underscores the need for an individualised management.

## Introduction

Sodium-glucose cotransporter 2 inhibitors (SGLT2i) are modern medications used in the treatment of type 2 diabetes, heart failure and chronic kidney disease. Their beneficial effects on cardiovascular and renal outcomes have been demonstrated in large clinical trials which led to a rapid increase in prescriptions in recent years.^1,2^ However, SGLT2i associated ketoacidosis – a rare but potentially life-threatening side effect – can occur. Current guidelines recommend the discontinuation of SGLT2i in the intensive care setting.^3,4^ However, in recent months, a number of studies on the use of SGLT2i in surgical and intensive care patients have been published that question these recommendations. A large retrospective cohort study from the United States revealed that while the incidence of SGLT2i associated ketoacidosis is significantly increased postoperatively compared with patients without SGLT2i, there are beneficial effects on kidney function and mortality.^5^ The authors concluded that a liberalisation of the current guidelines regarding the preoperative discontinuation of SGLT2i may be considered. Additionally, a prospective cohort study showed that initiation of SGLT2i therapy perioperatively is associated with a lower incidence of acute kidney injury in cardiac surgery patients suggesting that prescription before surgery might be indicated.^6^ While this trend is acknowledged, concerns remain regarding the occurrence of severe SGLT2i associated ketoacidosis, which could become more frequent if the current guidelines are relaxed. Against this background, we present a case that highlights the dangers of continuing SGLT2i treatment and examines several aspects of diagnosis and treatment of SGLT2i associated ketoacidosis in the intensive care setting with a special focus on the initiation of mechanical ventilation.

## Case presentation

The 63-year-old male patient was treated in May 2025 at the University Hospital Duesseldorf. He gave written informed consent for the presentation and publication of this case. He had a complex cardiac history, including severe pulmonary arterial hypertension and impaired right ventricular function, and was admitted with progressively worsening dyspnoea classified as NYHA IV. His medical background included three-vessel coronary artery disease, type 2 diabetes mellitus, for which he was taking 10 mg of dapagliflozin, and severe tricuspid valve insufficiency. At presentation, the patient reported increasing shortness of breath. Microbiological testing revealed the presence of *Escherichia coli* in both blood and urine cultures, prompting the initiation of broad-spectrum antibiotic therapy with piperacillin/tazobactam. On admission to the emergency department, the patient's kidney function was mildly impaired (creatinine 1.21 mg dl^−1^), but creatinine levels normalised over the course of hospitalisation. Despite treatment, the patient's clinical condition deteriorated further within the next days, marked by severe hypoxaemia. Antibiotic therapy was adjusted to levofloxacin in response to the evolving clinical picture of suspected pneumonia, and the patient was transferred to the intensive care unit (ICU) for closer monitoring and advanced support. At this point, he met all the diagnostic criteria for sepsis. Initial arterial blood gas analysis showed a base excess of −9.1 and a bicarbonate of 12.4 mmol l^−1^ with pH compensated by severe hyperventilation (see Table 1). Noninvasive ventilation was commenced to manage his severe hypoxaemia (see Table 1). Despite repeated administration of sodium bicarbonate, metabolic acidosis remained refractory. The patient's condition then deteriorated rapidly, necessitating endotracheal intubation and invasive mechanical ventilation. Following intubation mechanical ventilation was undertaken with standard settings. This way, pCO_2_ increased from 17.7 to 44.8 mmHg and the next arterial blood gas analysis unmasked the metabolic acidosis, with a drop in pH to 7.13 and a base excess of –14.5 mmol l^−1^. After induction of general anaesthesia for intubation and invasive ventilation, haemodynamic variables remained stable initially but, following the acid–base imbalance, escalating doses of catecholamines were required (norepinephrine at 1.17 μg kg^−1^ min^−1^, epinephrine at 0.12 μg kg^−1^ min^−1^, and vasopressin at 1.6 IU h^−1^). Shortly thereafter, the patient suffered from repeated episodes of ventricular tachycardia, necessitating cardiopulmonary resuscitation (CPR) including defibrillation. CPR time in total was 2 min. Given the rapid progression of haemodynamic instability, a multidisciplinary team promptly decided to initiate veno-arterial extracorporeal membrane oxygenation (VA-ECMO). The cardiorespiratory situation stabilised immediately after VA-ECMO implantation. As the patient's haemodynamic decompensation was primarily driven by severe acidosis, with pulmonary function largely preserved, ECMO sweep gas flow and FiO_2_ were set to 2 l min^−1^ and 0.8, respectively, while total blood flow was maintained at 6.4 l min^−1^.

**Table 1:** Blood gas analyses

		Pre VA-ECMO		Post VA-ECMO		
		00 : 00	01 : 00	04 : 00	06 : 00	08 : 00
pO_2_	mmHg	57.5	102	112	172	161
sO_2_	%	90	94	98	100	100
pCO_2_	mmHg	17.7	44.8	27.4	41.7	36
pH		7.459	7.130	7.277	7.310	7.432
HCO_3_^−^	mmol l^−1^	12.4	14.5	14.3	21.7	24.5
BE	mmol l^−1^	−9.1	−14.5	−17.1	−4.3	−0.2
Lac	mmol l^−1^	2.1	3.1	8.8	10.8	13.2
Na^+^	mmol l^−1^	136	139	135	144	144
K^+^	mmol l^−1^	4.6	4.8	5.0	3.6	3.8
Ca^2+^	mmol l^−1^	1.15	1.31	1.24	1.19	1.14
Glu	mg dl^−1^	209	255	318	333	345
Hb	mg dl^−1^	13.6	13.9	9.3	10.3	10.8
Ketones	mmol l^−1^			3.1		

BE, base excess; Ca^2+^, calcium; Glu, glucose; Hb, haemoglobin; HCO_3_, bicarbonate; K^+^, potassium; Lac, lactate; Na^+^, sodium; pCO_2_, partial carbon dioxide pressure; pO_2_, partial oxygen pressure; sO_2_, oxygen saturation.

Pre VA ECMO (00 : 00), mechanical ventilation was initiated to correct the hypoxaemia and hypercarbia. At (01 : 00) and CO_2_ was raised, revealing the severe underlying metabolic acidosis. Post VA-ECMO, metabolic imbalances gradually corrected themselves with treatment.

However, despite stabilisation, the patient's base excess remained profoundly negative, finally prompting measurement of serum ketone levels. This revealed significant ketonaemia (3.1 mmol l^−1^), leading to the diagnosis of SGLT2i associated ketoacidosis, probably triggered by the ongoing infection on the basis of continuous daily treatment with dapagliflozin. Subsequent treatment with glucose infusion and insulin therapy was initiated. Over the next 4 h, the patient's acid-base status improved, with normalisation of pH and base excess, and he remained haemodynamically stable under VA-ECMO support. We were able to extubate the patient's trachea 34 h after ECMO commencement. During this phase, the patient was fully conscious and had no neurological impairment. Although initial VA-ECMO weaning was successful, the patient subsequently developed recurrent right-heart failure and ultimately died in-hospital.

## Discussion

Our case report carries two main educational points:

(1) Unmasking severe SGLT2i-associated ketoacidosis after removal of respiratory compensation during induction of mechanical ventilation; a physiologically plausible but rarely documented mechanism.
(2) Continuation of SGLT2i therapy in the ICU setting or perioperatively, which is highly debated at the moment, still precipitates life-threatening metabolic derangements, particularly under septic conditions or cardiac compromise.

In the context of SGLT2i therapy, this is the first case reporting an association between SGLT2i associated ketoacidosis and the need for mechanical circulatory support. However, similar cases have already been reported independently of SGLT2i drug medication.^7,8^

## Conclusion

SGLT2i associated ketoacidosis remains a diagnostic challenge. This case highlights the particular danger of missing the diagnosis, especially in patients who present with another clear diagnoses, e.g. sepsis or cardiac failure. In these patients, metabolic acidosis can be explained by the underlying disease and the additional diagnosis of ketoacidosis may be missed. Therefore, treatment strategies may not include interventions to treat the SGLT2i associated ketoacidosis. The effect of rapid normalisation of pCO_2_, in patients in whom severe metabolic acidosis is compensated by hyperventilation can unmask the metabolic acidosis. This is most important in patients with cardiac failure and those with right ventricular failure because these patients may decompensate rapidly due to increased pulmonary artery pressures caused by acidosis. To avoid this problem, either the acid-base balance can be corrected before induction of anaesthesia or alternatively, e.g. in emergency situations, mechanical ventilation with (mild) hyperventilation can be continued after intubation to maintain acid-base balance until metabolic acidosis can be corrected. Although the temporal association between ongoing SGLT2i therapy and the development of ketoacidosis in this case is strong, concomitant sepsis likely acted as a major trigger, and thus a multifactorial origin of the metabolic derangement must be assumed. Our report therefore does not claim a direct causal relationship but highlights the possible contributory role of continued SGLT2i therapy under septic conditions. We acknowledge that this is a single case and therefore cannot establish generalisable conclusions; however, it illustrates a plausible clinical scenario that may become more frequent with increasing SGLT2i use in high-risk populations. This concern is supported by data from a recent retrospective cohort study by Iwasaki *et al.*, which found that preoperative use of SGLT2 inhibitors was significantly associated with a higher incidence of perioperative metabolic acidosis in euglycemic patients.^9^

Management of SGLT2i associated ketoacidosis in general follows the principles of diabetic ketoacidosis: fluid and electrolyte resuscitation, insulin infusion with glucose supplementation, and careful monitoring of potassium balance. Sodium bicarbonate may be considered in cases of severe acidaemia (pH ≤ 7.1), though its effect may be transient as, in contrast to glucose/insulin treatment, sodium bicarbonate does not correct the underlying abnormal physiology. Early ketone measurement in any patient on SGLT2i with metabolic acidosis is essential to avoid delayed diagnosis. Point-of-care tests measuring beta-hydroxybutyrate should be preferred over urine methods measuring only acetoacetate. Preventive strategies include withholding SGLT2i during acute illness, fasting, or sepsis, and at least three days before elective surgery. Nevertheless, emerging retrospective data suggest that universal discontinuation may not be appropriate for all patient populations, for example not for those with heart failure.^10^ Until prospective trials provide more clarity, individualised decision-making and multidisciplinary collaboration remain central to balance risks and benefits of SGLT2i therapy. Regardless of this, severe acidosis should always be avoided. As long as the awareness of SGLT2i associated ketoacidosis remains low, liberalising the current guidelines does not seem appropriate.
